# Ocular Manifestations of Systemic Diseases

**DOI:** 10.1155/2018/7851691

**Published:** 2018-10-24

**Authors:** Anna K. Nowinska, Anna Machalińska, László Módis, Robert Koprowski, Miguel Rechichi

**Affiliations:** ^1^Chair and Department of Ophthalmology, School of Medicine with the Division of Dentistry in Zabrze, Medical University of Silesia, Katowice, Poland; ^2^First Department of Ophthalmology, Pomeranian Medical University, Szczecin, Poland; ^3^Department of Ophthalmology, University of Debrecen, Budapest, Hungary; ^4^Department of Biomedical Computer Systems, University of Silesia, Faculty of Computer Science and Materials Science, Institute of Computer Science, Sosnowiec, Poland; ^5^Centro Polispecialistico Mediterraneo Via Chiubica SNC, Sellia Marina, 88050, Italy

Ocular examination is an essential diagnostic component of any physical examination, which can assist in differential diagnosis and be crucial in decision regarding the treatment method.

A significant number of diseases are known to present with ocular involvement, such as vascular diseases, diabetes mellitus, diseases involving nervous system, or rheumatoid diseases. Sometimes, ocular involvement is present as a primary symptom, such as peripheral corneal ulcer in the case of Wegener granulomatosis or rheumatoid arthritis, or ocular involvement is secondary to systemic diseases, for example, retinal angiopathy related to hypertension.

Ocular symptoms connected to systemic diseases involve both anterior and posterior eye segments and all tissues of the eye globe. Therefore, there is a wide variety of ocular symptoms in clinical practice which are crucial in differential diagnosis.

Recent advances in diagnostic techniques in ophthalmology, for example, optical coherence tomography angiography (OCTA), give new opportunities to diagnose microvascular systemic changes.

Authors contributed a range of papers including 2 clinical studies, 6 research articles, and 1 review.

A brief description of these 9 works is as follows:

1. The clinical study “Choroid and Retinal Nerve Fiber Layer Thickness in Patients with Chronic Obstructive Pulmonary Disease Exacerbation” revealed decreased subfoveal choroid thickness in the COPD patients both during an exacerbation and in the stable period. Since the normal choroidal vasculature is essential for retinal function, thinning of the choroid and loss of the vascular tissues could lead to photoreceptor damage and vascular dysfunction in such patients.
2. The authors of the research article “Age Differences in Axial Length, Corneal Curvature, and Corneal Astigmatism in Marfan Syndrome with Ectopia Lentis” studied ocular parameters in patients with Marfan syndrome and ectopia lentis. They observed that axial length varies with age, corneal curvature remains stable, and corneal astigmatism is higher in young patients and tends to shift toward against-the-rule or oblique astigmatism. Therefore, it is important to consider age when diagnosing MFS with ocular biometric data.
3. The paper written by João Beirão and colleagues assessed the aqueous humor flare in transthyretin V30M amyloidosis patients (ATTRV30M). The authors revealed that the aqueous humor flare values in the scalloped iris eyes may be a valid marker for controlling the stage of the oculopathy in ATTRV30M patients. What is worth underlining, the flare values suggest that controlling the stage and progression of glaucoma might be key in the surveillance scalloped iris eyes in ATTRV30M patients and may be considered as an evaluation method of future treatments.
4. The authors of the research article “*HLA-C* Alleles and Cytomegalovirus Retinitis in Brazilian Patients with AIDS” typed *HLA-C* locus in patients with AIDS exhibiting or not cytomegalovirus retinitis (CR). Based on the 412 patients and controls studied, they revealed that the *HLA-C∗07* allele group conferred protection against the development of CR in Brazilian AIDS patients, whereas the *HLA-C∗05* and *HLA-C∗16* allele groups were associated with AIDS susceptibility and protection, respectively.
5. The paper “Clinical and Genetic Features of Tubulointerstitial Nephritis and Uveitis Syndrome with Long-Term Follow-Up” focuses on the clinical manifestations, prognosis, and HLA type of tubulointerstitial nephritis and uveitis syndrome (TINU). TINU is a rare, specific form of intraocular inflammation (uveitis) combined with kidney disease that affects approximately 1-2% of the uveitis patients. The authors studied 5 cases of TINU with a mean age of 15.8 years and a mean follow-up of 54.0 months. Since the differential diagnosis of the disease is challenging, the authors suggest that the urinary *β*2 microglobulin level and HLA typing may help in the diagnosis process.
6. Per Kappelgaard and colleagues published a novelty result based on their clinical study on “Retinal Vessel Diameter Changes in Relation to Dark Adaptation and Acute Hyperglycemia.” The diabetes mellitus and hyperglycemia is an increasing and challenging general health problem; thus, understanding the pathogenesis of the diabetic retinopathy seems to be a very important and urgent issue. The authors revealed that darkness and fasting were both associated with retinal vasodilation in patients with type 2 diabetes. They also suggest that future studies should determine whether both of the two stimuli of vasodilation lead to retinal hyperperfusion, which would support that they may be involved in the aggravation of diabetic retinopathy.
7. The review article “Ocular Manifestations of Alzheimer's and Other Neurodegenerative Diseases: The Prospect of the Eye as a Tool for the Early Diagnosis of Alzheimer's Disease” presents retinal findings specific for neurodegenerative diseases, such as *β*-amyloid plaques in retina tissue (including the RGC, retinal nerve fibre layer (RNFL), and inner plexiform layer), which can produce a fluorescence effect by using curcumin as a contrast.
8. The authors of the paper “Correlation between CHA_2_DS_2_-VASc Score and Glaucoma Treatment and Prognosis” used the CHA_2_DS_2_-VASc score which was first developed by cardiologists to assess the need of anticoagulant therapy after the detection of atrial fibrillation (AF) to assess the risk of glaucoma progression. They revealed that higher CHA2DS2-VASc scores correlated with the need of more aggressive treatment chosen from either monotherapy, dual therapy, laser treatment, or surgery treatment.
9. The research article “Vision-Threatening Behçet's Disease: Severity of Ocular Involvement Predictors” addressed the problem of Behçet's disease visual acuity prognosis for patients. The authors studied several general findings: systemic vasculitis and oral and genital ulcers whose occurrence and severity could potentially help ophthalmologists categorize their patients based on future risk and treatment plan accordingly.

We would like to extend our gratitude to all the authors who submitted their work for consideration in our special issue and to the reviewers for their critical feedback. We hope that this collection of works provides a new insight into diagnostic and treatment methods of ocular disorders connected to systemic diseases.

